# A Comparative Study of Extended Gentamicin and Tobramycin Release and Antibacterial Efficacy from Palacos and Simplex Acrylic Cements

**DOI:** 10.3390/microorganisms13092174

**Published:** 2025-09-17

**Authors:** Débora Coraça-Huber, Martina Humez, Klaus-Dieter Kühn

**Affiliations:** 1 Research Laboratory for Biofilms and Implant Associated Infections (Biofilm-Infection Lab), Experimental Orthopaedics, University Hospital for Orthopaedics and Traumatology, Medical University of Innsbruck, Müllerstrasse 44, 6020 Innsbruck, Austria; debora.coraca-huber@i-med.ac.at; 2 Institute of Microbiology and Molecular Biology, Justus-Liebig-Universität Giessen, Heinrich-Buff-Ring 26, 35392 Giessen, Germany; 3 Heraeus Medical GmbH, Philipp-Reis-Straße 8-13, 61273 Wehrheim, Germany; 4 Department of Orthopaedics and Trauma, Medical University of Graz, Auenbruggerplatz 5, 8036 Graz, Austria

**Keywords:** antibiotic loaded bone cements (ALBCs), Simplex T, Palacos R+G, aminoglycoside, antimicrobial efficacy, HPLC elution, inhibition zone test

## Abstract

Antibiotic-loaded bone cements (ALBCs) are used to prevent and treat periprosthetic joint infections (PJI). This study compares the in vitro release and antibacterial effectiveness of gentamicin from Palacos^®^ R+G and tobramycin from Simplex^®^ T. Standardized cylindrical specimens of Palacos^®^ R+G and Simplex^®^ T were incubated in phosphate-buffered saline. Antibiotic release was quantified using high-performance liquid chromatography (HPLC) over 14 and 42 days. Antibacterial efficacy was assessed using inhibition zone tests (IZT) against *Staphylococcus aureus*, *Staphylococcus epidermidis*, and *Escherichia coli* over 42 days. Palacos^®^ R+G exhibited a significantly higher and more sustained antibiotic release of gentamicin compared to tobramycin from Simplex^®^ T. The cumulative release of gentamicin from Palacos^®^ R+G was 1148.00 µg/cm^2^, while Simplex^®^ T released 198.87 µg/cm^2^ tobramycin over 14 days. Inhibition zone tests showed that Palacos^®^ R+G maintained antibacterial activity for 42 days, while Simplex^®^ T’s activity diminished after 14 days. Statistical analysis confirmed significant differences in antibacterial efficacy between the two cements. Palacos^®^ R+G demonstrated superior gentamicin release and sustained antibacterial activity compared to tobramycin from Simplex^®^ T. These findings suggest that Palacos^®^ R+G may offer better clinical outcomes in preventing and treating PJIs.

## 1. Introduction

Polymethylmethacrylate (PMMA) bone cements are extensively utilized to anchor artificial joints. However, periprosthetic joint infections (PJI) remain a significant and devastating complication [1]. The treatment of joint infections poses a major and demanding challenge for both patients and surgeons [2]. Antibiotic-loaded bone cements (ALBCs) release antibiotics locally, thereby protecting the surgical field. In primary surgical procedures, ALBCs prevent biofilm formation on prostheses and significantly reduce the risk of infection within the joint [3]. In septic revisions, ALBCs serve as an adjuvant therapy to support bacterial eradication [4], effectively securing surgical debridement [5,6,7]. In two-stage revisions, antibiotic-loaded PMMA spacers are typically used in the interim phase. These spacers maintain a certain amount of joint stability and mobility and ensure high intra-articular antibiotic concentrations [8]. It is often recommended to use a combination of at least two antibiotics for spacer preparation: a broad-spectrum antibiotic (e.g., gentamicin), in combination with another antibiotic targeting the relevant pathogens [9,10]. According to the patient’s antibiogram, antibiotics can be manually added to the PMMA cement spacer if needed [11]. Admixing antibiotics to commercially available cements requires the consideration of factors such as the influence of the antibiotic amount on both antibiotic elution and mechanical stability [12,13].

The determination of antibiotic elution from PMMA is crucial for evaluating the efficacy of these cement spacers. Various methods, including enzyme-linked immunosorbent assay (ELISA), inhibition zone test (IZT), agar diffusion test, high-performance liquid chromatography (HPLC), and proliferation assays, are employed to measure antibiotic release [14,15,16,17,18,19,20,21]. The literature reveals notable differences in results depending on the method used. Even the decision on a medium influences bacterial growth and therefore also the antimicrobial efficacy of the test samples [22,23]. For instance, a recent publication using ELISA [17] demonstrated significantly higher release rates for the Cemex^®^ spacer (Tecres, Sommacampagna, Italy) to Refobacin^®^ Bone Cement spacers (Zimmer Biomet, Warsaw, IN, USA). Conversely, studies utilizing the IZT method indicated that the Cemex^®^ spacer had a lower release kinetic than the Copal^®^ Exchange spacer [13]. Furthermore, HPLC-based analyses presented yet another set of differing results. Unfortunately, there is no uniform, standardized procedure for determining the efficacy and especially the elution of antibiotics from ALBCs [24], making it difficult to compare most publications in this field.

In addition to HPLC/LC-MS/MS and inhibition zone bioassays (IZT/agar diffusion), other methods for quantifying antibiotic elution from PMMA have been described in the literature: (i) UV-Vis spectrophotometry (particularly for tobramycin, λ ≈ 269 nm) as a rapid, low-cost screening method, (ii) colorimetric ninhydrin assays or fluorescence polarization immunoassays for gentamicin, (iii) isothermal microcalorimetry to assess functional activity against (biofilm) bacteria on or near the PMMA surface, (iv) broth microdilution or time-kill assays using eluted samples, and (v) in vivo/intra-articular micro dialysis to characterize early elution kinetics after spacer implantation. Each method has specific strengths and limitations in terms of sensitivity, matrix interference, and clinical applicability [25].

These discrepancies highlight the importance of not only the methods themselves but also factors such as sample preparation and the concentration and quality of active substances [10,26,27]. Other factors include the thermostability of the incorporated antibiotics [26], the cement surface area [2,28] and the ability of the PMMA cement to absorb water, which correlates with the polymer base and the hydrophilicity of the PMMA cement [2,29]. The more water the cement matrix can absorb, the more antibiotic can be eluted. PMMA cements containing hydrophobic styrene result in lower hydrophilicity compared to pure PMMA-MMA copolymer cements [30]. Therefore, the antibiotic elution varies by cement type, with Simplex^®^ P exhibiting the lowest release compared to Palacos^®^ and Copal^®^ formulations [31,32]. Even the mixing procedure influences antibiotic elution, depending on the cement porosity. A decrease in porosity due to vacuum mixing decreases antibiotic elution [29], whereas an increase in cement porosity by adding pore-induced particles increases the elution but decreases the mechanical properties significantly. The variability in these factors makes direct comparisons between studies challenging.

We aim to prove that the standardized application of HPLC and IZT for determining antibiotic elution from various ALBC formulations enables the consistent and comparable assessment of their antimicrobial efficacy, regardless of cement type and antibiotic concentration. We hypothesize that the temporal profile and total amount of antibiotic release differ significantly between commercially available bone cement formulations. 

## 2. Materials and Methods

### 2.1. Bone Cements and Bacterial Strains

Two commercially available aminoglycoside-containing bone cements were used in this study; Palacos^®^ R+G, containing 0.5 g gentamicin (Heraeus Medical GmbH, Wehrheim, Germany), and Simplex^®^ T (also branded as Simplex^®^ P with Tobramycin) with 1.0 g tobramycin (Stryker, Kalamazoo, MI, USA) (Table 1). Both cements were prepared according to the manufacturers’ instructions for use [33,34] using the Palamix^®^ Mixing Device (Heraeus Medical GmbH, Wehrheim, Germany) without vacuum, and shaped into standardized cylindrical specimens with a diameter of 25 mm and a height of 10 mm. Phosphate-buffered saline (PBS, pH 7.4) was used as the incubation medium for the antibiotic release and inhibition zone tests. Mueller–Hinton agar (Oxoid, ThermoFisher Scientific, Darmstadt, Germany) was used for antimicrobial susceptibility testing [35]. Reference bacterial strains from the DSMZ collection were employed; *Staphylococcus aureus* DSM 799 (ATCC 6538), *Staphylococcus epidermidis* DSM 1798, and *Escherichia coli* DSM 1576 (ATCC 8739) (Table 2), all susceptible against aminoglycoside antibiotics gentamicin and tobramycin. Both antibiotics are comparable in their antimicrobial spectrum as well as mode of action. All agar-based experiments followed the EUCAST 2025 [36] guidelines for antimicrobial testing.

According to EUCAST 2025 [37], aminoglycoside breakpoints relevant to our study are as follows: Enterobacterales (including *E. coli*) are generally considered susceptible at MIC ≤ 2 mg/L for both gentamicin and tobramycin [38]; for staphylococci, gentamicin breakpoints are reported mainly for synergistic therapy. Thus, both gentamicin and tobramycin are in principle active against the reference strains used. Our E-test results confirmed this for *S. aureus* (MIC gentamicin 0.25 µg/mL) and *S. epidermidis* (0.125 µg/mL); the IZT data further showed inhibition zones against *E. coli*, confirming the biological activity of the antibiotics eluted from the cement.

### 2.2. Short- and Long-Term Release Rate of Gentamicin and Tobramycin

Standardized cylindrical bone cement specimens (25 mm diameter, 10 mm height, surface area: 8.0 cm^2^) were incubated at 37 °C in 10.5 mL of dissolution medium (0.1 M phosphate buffer, pH 7.4). At pre-defined time points (for short term release 6 h, 12 h, 24 h, and for long term release 1 d, 3 d, 7 d, 14 d, 28 d, 42 d), aliquots were collected and the medium was fully renewed. All samples were stored at −20 °C until analysis. Calibration standards were prepared in the following concentration ranges: 100–7500 ng/mL for gentamicin, 50–2500 ng/mL for tobramycin. Each set included a zero sample (without internal standard) and a blank sample (with internal standard). To prepare the standards, 200 µL of working solution was spiked with 18 µL of the corresponding internal standard solution (gentamicin and tobramycin). These solutions were used for LC-MS/MS analysis (AZ Biopharm, Berlin, Germany). Study samples were diluted 20-fold for gentamicin and 5-fold for tobramycin and processed using the same protocol as for the calibration standards. Internal standard working solutions were added accordingly. All samples were analyzed in triplicate. Chromatographic separation was performed using a modular HPLC 1200 Series system (Agilent Technologies, Waldbronn, Germany) equipped with a Luna C18(2) column (150 × 2 mm) and two C18 guard columns (4 × 2 mm; Phenomenex, Aschaffenburg, Germany). Column temperature was maintained at 25 °C. The injection volume was 2 µL. Mobile phase A consisted of 0.11 M trifluoroacetic acid/methanol (50:50), and mobile phase B was acetonitrile. Isocratic separation was achieved at an A:B ratio of 95:5, with a flow rate of 0.25 mL/min. The run time was 2.5 min, and the total cycle time was less than 3 min. Under these conditions, the four gentamicin components (C1, C2, C2a, and C1a) were co-eluted. This HPLC method was previously described by Heller et al. [39] for quantifying gentamicin in biopsy samples. Detection was performed on an API 4000 QTrap mass spectrometer (Applied Biosystems, Darmstadt, Germany) equipped with an electrospray ionization source operating in positive polarity and multiple reaction monitoring (MRM) mode. The following MRM transitions were monitored: 478.4 → 322.3 *m/z* (gentamicin C1), 464.4 → 322.3 *m/z* (gentamicin C2 and C2a), 450.3 → 322.3 *m/z* (gentamicin C1a), and 468.4 → 163.1 *m/z* (internal standard). The summed peak areas of the gentamicin components were analyzed using Analyst software version 1.4.2 (Applied Biosystems, ThermoFisher Scientific, Darmstadt, Germany), and data were processed with Microsoft Excel 365 (Microsoft Deutschland GmbH, Unterschleißheim, Germany). Tobramycin and its internal standard were analyzed under the same LC-MS/MS conditions. The MRM transitions monitored were 163.1 *m/z* for tobramycin and 478.4 → 322.3 *m/z* for its internal standard. Chromatograms were evaluated using Analyst 1.4.2 (AB Sciex LLC., Framingham, MA, USA) and processed with Microsoft Excel 365.

### 2.3. Comparative Long-Term Efficacy of Gentamicin and Tobramycin

The antibacterial efficacy of gentamicin and tobramycin released from the two bone cements tested was evaluated using the IZT method. The following reference strains from the DSMZ collection were used: *Staphylococcus aureus* DSM 799, *Staphylococcus epidermidis* DSM 1798 and *Escherichia coli* DSM 1576. The bacterial strains were initially tested for their susceptibility to gentamicin and tobramycin using E-tests (BioMérieux GmbH, Vienna, Austria). Each E-test was performed in triplicate. The minimum inhibitory concentration (MIC) values for the tested antibiotics were determined to be 0.25 μg/mL for *S. aureus*, 0.125 μg/mL for *S. epidermidis* and 0.125 µg/mL for *E. coli*. All antimicrobial susceptibility tests were performed on Mueller–Hinton agar according to EUCAST 2025 guidelines [36], using standardized agar volumes. Plates were incubated at 36 ± 1 °C for 24 h. To assess the release efficacy of gentamicin from Palacos^®^ R+G and tobramycin from Simplex^®^ T cements, standardized cylindrical specimens (25 mm diameter × 10 mm height) were used. One cement body per group was incubated in phosphate-buffered saline (PBS) at room temperature. Sampling was performed at the following time points: 1 h, 24 h, 7 d, 14 d, 21 d, 35 d, 42 d. At each time point, 60 μL of the incubation solution was withdrawn and applied directly onto Mueller–Hinton agar plates previously inoculated with one of the bacterial strains and pre-incubated for 24 h at 37 °C. For each time point and cement type, three independent cement specimens were tested (n = 3). The diameter of the inhibition zones was measured (in mm) for each of the tested bacterial strains (*S. aureus*, *S. epidermidis*, and *E. coli*). Mean values and standard deviations were calculated for each group. For all elution and efficacy tests, we used cement samples that were prepared in a standardized way, ensuring a defined surface.

### 2.4. Statistical Analysis

To evaluate the differences in antimicrobial activity between Palacos^®^ R+G and Simplex^®^ T, statistical analysis was performed on the inhibition zone diameters obtained for *Staphylococcus aureus*, *Staphylococcus epidermidis*, and *Escherichia coli*. Data were collected at seven time points over a 42-day period. The mean inhibition zones were compared using an unpaired two-tailed Student’s *t*-test assuming unequal variances. Additionally, two-way ANOVA (factors: cement type and time), followed by Bonferroni-corrected post hoc pairwise comparison, was performed to confirm the statistical significance of the observed differences between groups. A significance threshold of *p* < 0.05 was adopted for all comparisons. Statistical analysis was conducted using Python version 3.12.10 and SciPy 1.16.0 with the SciPy library (NumFOCUS, Austin, TX, USA).

## 3. Results

### 3.1. Comparative Short-Term Release Rate of Gentamicin and Tobramycin

**Figure 1:**
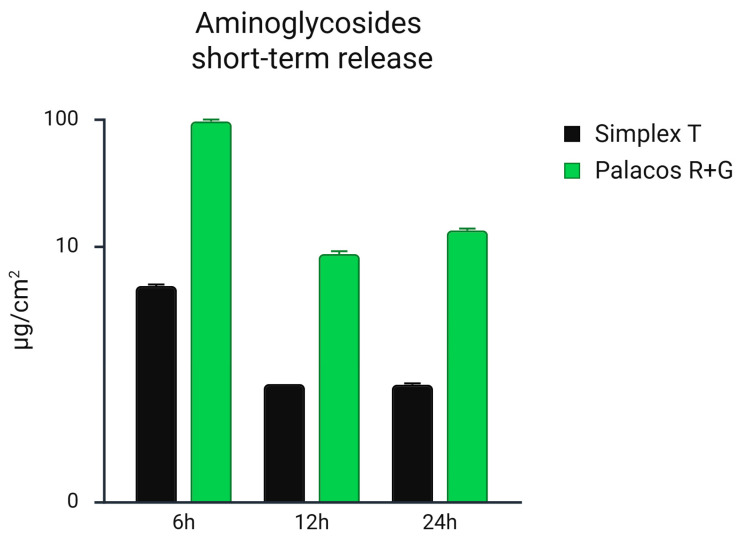
Short-term release of gentamicin from Palacos^®^ R+G (1.25% gentamicin) and tobramycin from Simplex^®^ T (2.5% tobramycin) bone cements over the first 24 h, quantified by HPLC and expressed in µg/cm^2^. Palacos^®^ R+G exhibited substantially higher and more sustained antibiotic release across all time points compared to Simplex^®^ T. Statistical analysis showed a significant difference in release profiles between the two materials over time (one-way ANOVA, *p* = 0.044) and a trend toward significance in direct group comparison (unpaired *t*-test, *p* = 0.059).

Tobramycin release from Simplex^®^ T: The cumulative release of tobramycin from the Simplex^®^ T bone cement was evaluated over a 24 h period (Figure 1). The initial burst release observed at 6 h reached a mean concentration of 4.83 µg/cm^2^ (SD: 0.09), representing the highest value within the sampling period. This was followed by a sharp decrease in concentration at 12 h and 24 h, reaching 0.74 µg/cm^2^ (SD: 0.02) and 0.73 µg/cm^2^ (SD: 0.01), respectively. Overall, the cumulative release across all time points for Simplex^®^ T resulted in a total mean tobramycin concentration of 7.30 µg/cm^2^ (SD: 0.52).

Gentamicin release from Palacos^®^ R+G: In contrast, Palacos^®^ R+G demonstrated a significantly higher gentamicin release profile (Figure 1). At 6 h, the release peaked at a mean of 96.00 µg/cm^2^ (SD: 2.3), nearly 20-fold higher than tobramycin from Simplex^®^ T at the same time point. Although there was a notable decrease at 12 h (8.70 µg/cm^2^, SD: 0.43), a secondary peak was observed at 24 h, reaching 13.30 µg/cm^2^ (SD: 0.33). Gentamicin concentrations remained consistently higher than those observed for tobramycin from Simplex^®^ T throughout the 24 h. The total cumulative gentamicin release from Palacos^®^ R+G over the entire period was 183.61 µg/cm^2^ (SD: 2.21), more than 25 times higher than that observed for tobramycin of Simplex^®^ T.

The quantification of gentamicin release via HPLC over the first 24 h revealed that Palacos^®^ R+G consistently released higher concentrations of antibiotic compared to tobramycin of Simplex^®^ T (Figure 1). At all measured time points, the gentamicin release from Palacos^®^ R+G remained substantially elevated, particularly in the early phase (e.g., 96.00 µg/cm^2^ at 6 h vs. 4.83 µg/cm^2^ for tobramycin of Simplex^®^ T). Statistical analysis using a one-way ANOVA confirmed a significant difference between the two cements (*p* = 0.044), indicating that the overall gentamicin release profile was higher in the Palacos^®^ R+G group with 1.25% gentamicin compared to Simplex^®^ T with 2.5% tobramycin. The unpaired t-test showed a trend toward significance (*p* = 0.059), suggesting variability but supporting the same directional outcome. These findings reinforce the enhanced early and intermediate-term antibiotic elution performance of Palacos^®^ R+G.

### 3.2. Comparative Long-Term Release Rate of Gentamicin and Tobramycin

**Figure 2:**
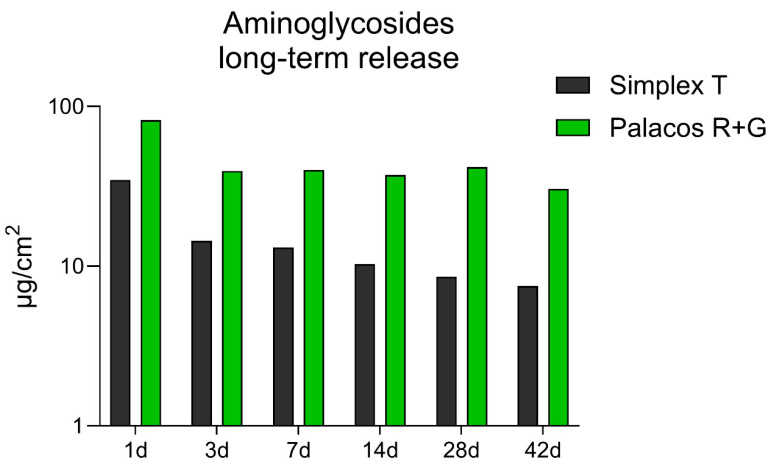
Long-term release of gentamicin from Palacos^®^ R+G (1.25% gentamicin) and tobramycin from Simplex^®^ T (2.5% tobramycin) bone cements, quantified by HPLC expressed in µg/cm^2^. Palacos^®^ R+G exhibited substantially higher and more sustained antibiotic release across all time points compared to Simplex^®^ T over a 42-day period; Palacos^®^ R+G demonstrated significantly higher and more sustained antibiotic release across all time points compared to Simplex^®^ T (*p* < 0.01, unpaired *t*-test and ANOVA).

The following data presents a direct comparison of gentamicin release profiles from Palacos^®^ R+G and Simplex^®^ T bone cements, measured in µg/cm^2^, over a 42-day period (Figure 2). Palacos^®^ R+G exhibited significantly higher antibiotic release across all time points. On day 1, Palacos^®^ R+G showed a peak release of 82.2 µg/cm^2^, more than double that of Simplex^®^ T, which released 35.6 µg/cm^2^. Although the release from both cements decreased over time, Palacos^®^ R+G maintained a more sustained release pattern. For instance, by day 14, the release values were 37.2 µg/cm^2^ for Palacos^®^ R+G and only 10.3 µg/cm^2^ for Simplex^®^ T. At the end of the study period (day 42), Palacos^®^ R+G continued to release gentamicin at 30.5 µg/cm^2^, while Simplex^®^ T showed a markedly lower release of 7.5 µg/cm^2^. These results emphasize the superior long-term elution characteristics of Palacos^®^ R+G, which may contribute to improved local antibiotic availability in clinical applications requiring extended prophylaxis or infection control.

### 3.3. Antibiotic Susceptibility Tests Carried Out During Comparative Long-Term Tests on Gentamicin and Tobramycin from Palacos^®^ R+G and Simplex^®^ T

**Figure 3:**
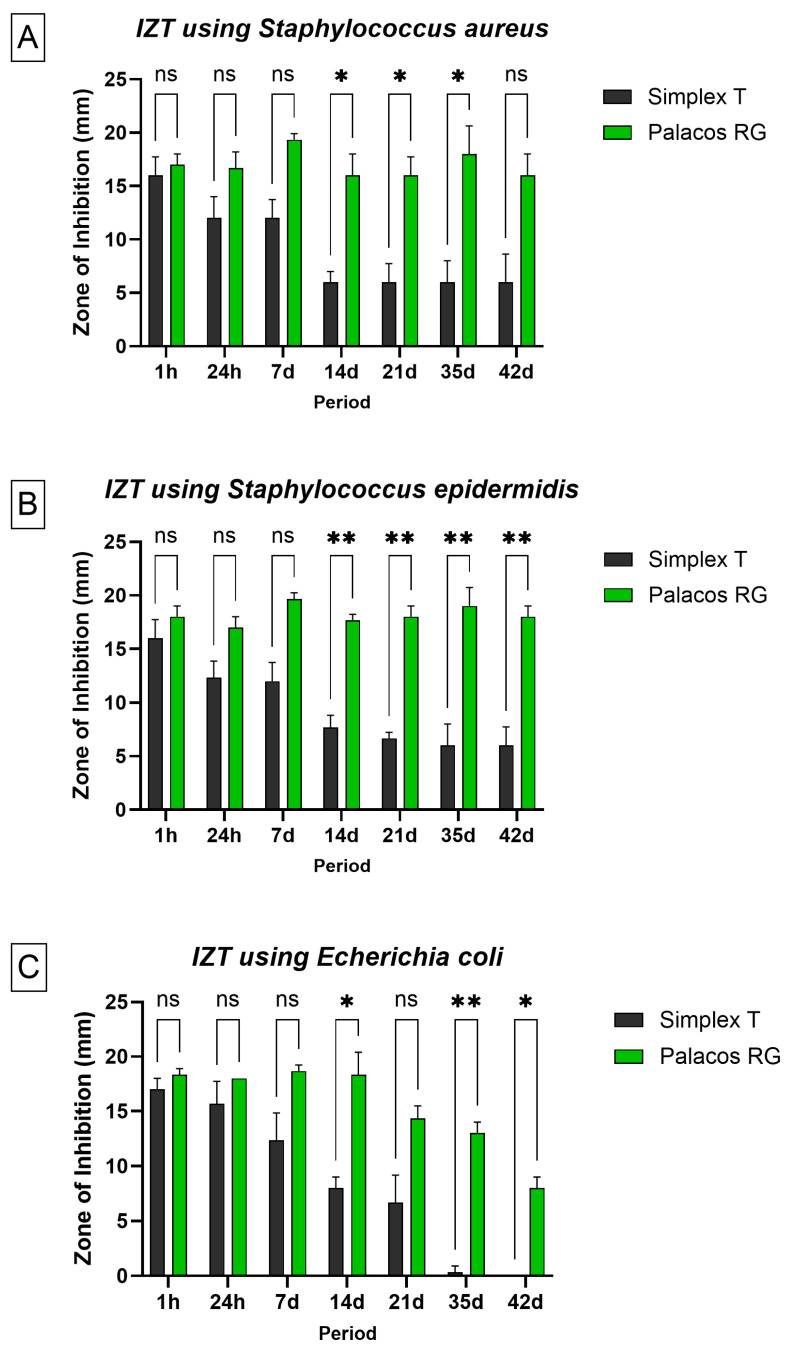
Inhibition zones (in mm) produced by eluates from Palacos^®^ R+G (1.25% gentamicin; green bars) and Simplex^®^ T (2.5% tobramycin; black bars) against three bacterial strains: (**A**) *Staphylococcus aureus*, (**B**) *Staphylococcus epidermidis*, and (**C**) *Escherichia coli*, measured at multiple time points (1 h to 42 days). Each bar represents the mean ± standard deviation (n = 3). Statistical analysis was performed using a two-way ANOVA (factors: cement type and time), followed by Bonferroni-corrected post hoc pairwise comparisons. Palacos^®^ R+G showed significantly larger inhibition zones than Simplex^®^ T at later time points for *S. aureus* (days 14, 32, 35; *p* < 0.05), *S. epidermidis* (days 14, 21, 35, 42 *p* ≤ 0.01), and *E. coli* (days 14, 35, 42; *p* < 0.05). ns = not significant; * = *p* < 0.05; ** = *p* ≤ 0.01.

The antimicrobial activity of the ALBCs was assessed by measuring the diameter of inhibition zones against *Staphylococcus aureus*, *Staphylococcus epidermidis*, and *Escherichia coli* over a period of 42 days (Supplementary Data S1). Measurements were taken after applying eluates from cement samples incubated in PBS to pre-inoculated Mueller–Hinton agar plates. Figure 3A shows the inhibition zones against *S. aureus*. Palacos^®^ R+G exhibited strong antibacterial activity at early time points, with a mean inhibition zone of approximately 24 mm at 1 h. This activity gradually decreased over time but remained detectable up to day 42. In contrast, Simplex^®^ T showed significantly smaller zones, with measurable activity only until day 14, and no inhibition observed thereafter. Figure 3B illustrates the results for *S. epidermidis*. A similar trend was observed: Palacos^®^ R+G showed robust antibacterial activity with inhibition zones above 20 mm in the first 24 h, which declined gradually but persisted throughout the 42-day period. Simplex^®^ T demonstrated limited activity, with inhibition zones significantly smaller than those of Palacos^®^ R+G and becoming undetectable by day 28. Figure 3C presents the inhibition data for *E. coli.* The activity of Palacos^®^ R+G was again superior, showing measurable inhibition up to 28 days, whereas Simplex^®^ T displayed minimal efficacy, with small inhibition zones only detectable up to 7 days. In all three bacterial models, Palacos^®^ R+G demonstrated superior and more sustained antibacterial activity compared to Simplex^®^ T. Palacos^®^ R+G showed significantly larger inhibition zones than Simplex^®^ T at later time points for *S. aureus* (days 14, 21, 35; *p* < 0.05), *S. epidermidis* (days 14, 21, 35, 42; *p* ≤ 0.01), and *E. coli* (days 14, 35, 42; *p* < 0.05). Early time points (1 h, 24 h, 7 d) did not show significant differences between cements. These results confirm that Palacos^®^ R+G exhibited superior and prolonged antibacterial efficacy compared to Simplex^®^ T, particularly at intermediate and late time points.

## 4. Discussion

The methodology used in this study, including HPLC and IZT, provides a comprehensive evaluation of antibiotic elution and efficacy. HPLC is a highly sensitive and specific method that allows for precise quantification of antibiotic concentrations over time [39]. This method is particularly useful for detecting subtle differences in elution profiles and ensuring accurate measurements of antibiotic release [39]. Kittinger et al., 2024 [15] stated that HPLC showed the best differences in release kinetics and helped to prove that the total amount of antibiotic added is not directly proportional to the antibiotic elution. In contrast, IZT assesses the antibacterial activity of the eluted antibiotics by measuring the diameter of inhibition zones against specific bacterial strains [19]. Methodologically, the parallel use of quantitative (HPLC/LC-MS/MS) and functional (IZT) assays provided complementary information: HPLC/LC-MS/MS yielded quantitative elution kinetics, whereas IZT reflected the biological activity of the antibiotic fractions that diffused into the agar. However, the combination of HPLC and IZT in this study offers a robust assessment of both the quantitative and qualitative aspects of antibiotic elution and efficacy [15]. Differences between the two methods arise from matrix binding, diffusion radii, and medium conditions (moisture/nutrients), and are therefore expected [15]. Elution data should thus always be interpreted in conjunction with activity assays. Pharmacologically, gentamicin and tobramycin share similar activity against Enterobacterales; for *Pseudomonas aeruginosa*, tobramycin has been reported to show slightly higher in vitro activity, while for Staphylococci, gentamicin is the more established aminoglycoside (usually in combination for synergistic effect) [40]. In our model (*E. coli*), both materials showed only limited activity over time, likely reflecting the free drug concentration achievable in the cement matrix rather than a fundamental class-spectrum difference.

Other methods frequently used to determine the antimicrobial efficacy of antibiotic-loaded bone cements include ELISA, agar diffusion test, and proliferation assays. Each method has its advantages and limitations. ELISA is highly sensitive and specific for detecting and quantifying proteins, including antibiotics [17]. It is particularly useful for measuring low concentrations of antibiotics in complex biological samples [19]. However, ELISA requires specific antibodies that are not available for all antibiotics. The agar diffusion test is like IZT, and it assesses the antibacterial activity of antibiotics by measuring the zones of inhibition on agar plates [19]. It is relatively simple and cost-effective but lacks the precision of HPLC in quantifying antibiotic concentrations. Also, it is not suitable for the determination of antibiotic elution from PMMA cement samples as it underestimates the actual antibiotic elution [15]. Additionally, EUCAST [36] does not recommend agar diffusion tests like Kirby–Bauer disk diffusion for routine antimicrobial susceptibility testing. Instead, EUCAST primarily endorses broth microdilution for MIC determination and standardized disk diffusion tests. The “pour plate” or “incorporation” method, which involves mixing bacteria into molten agar, is not recommended due to its lack of standardization and reproducibility. This method is more suitable for testing bacteriophage or bacteriocin activity, some biocompatibility assays, and specific microbiology applications. Proliferation assays measure the effect of antibiotics on bacterial growth and proliferation. They provide valuable information on the biological efficacy of antibiotics, especially when combined with other testing methods, e.g., IZT or an in vivo biofilm model [41].

The composition of nutrient media and environmental conditions such as humidity significantly influence antibiotic activity and the outcomes of antimicrobial susceptibility testing. Variations in media components can affect bacterial growth, biofilm formation, and the expression of resistance genes, leading to differences in observed antibiotic efficacy [22,23,35]. Excess surface moisture can cause the irregular diffusion of antibiotics, resulting in larger and inconsistent inhibition zones. Conversely, a dry agar surface can limit antibiotic diffusion, leading to smaller inhibition zones and potentially false resistance results. High incubation humidity helps maintain standard diffusion dynamics by preventing the agar from drying out [22,23,35].

Given the variability in these factors, the objective of this study is to provide a comprehensive recommendation for the determination of antibiotic elution using HPLC and IZT. By standardizing these methods, we aim to facilitate more consistent and comparable evaluations of ALBC efficacy. The cements used in this study were different in composition and drug concentration, and the cement sample preparation involved standardized molded bodies with a defined surface. The same eluates were used for all efficacy tests to ensure uniformity.

The results of this study demonstrate a significant difference in the antibiotic elution profiles between Palacos^®^ R+G and Simplex^®^ T bone cements. Over a 14-day period, Palacos^®^ R+G demonstrated significantly enhanced and prolonged aminoglycoside release compared to Simplex^®^ T, despite containing only 0.5 g of gentamicin, whereas Simplex^®^ T incorporates 1 g of tobramycin. This difference was particularly pronounced in the early phase, with Palacos^®^ R+G showing a peak release nearly 20-fold higher than Simplex^®^ T at the same time point. The IZT further corroborated these findings, showing that Palacos^®^ R+G maintained antibacterial activity for up to 42 days, while Simplex^®^ T’s activity diminished after 14 days. The superior elution profile of Palacos^®^ R+G can be attributed to its formulation, which likely facilitates better antibiotic release and sustained antibacterial efficacy [30,42]. These results suggest that Palacos^®^ R+G may offer better clinical outcomes in preventing and treating PJI due to its enhanced antibiotic elution and prolonged antibacterial activity. This finding is in line with the in vitro testing from Meeker et al., 2019 [31] that found that the elution profiles of vancomycin, daptomycin and tobramycin manually added to Simplex^®^ and Palacos^®^ bone cements were higher for the latter one. Despite Dietz et al., 2024 [43]’s finding of a high antibiotic release rate for Simplex^®^ T on day 1, the decrease on day 2 was massive compared to Palacos^®^ R+G. After day 1, Simplex^®^ T did not even reach the MIC of the tested microorganisms, in contrast to Palacos^®^ R+G cements. Although gentamicin and tobramycin exhibit minor structural differences, both aminoglycoside antibiotics share a comparable mode of action and display a similar antimicrobial spectrum [44,45]. While tobramycin is often considered more effective against Gram-negative bacilli, the existing literature also highlights that a substantial proportion of these pathogens remain susceptible to gentamicin, underscoring its continued clinical relevance [45,46]. The primary distinction lies in tobramycin’s enhanced activity against *Pseudomonas aeruginosa* [45,47], a pathogen that, however, accounts for only 3–5% of all PJIs [48]. Tobramycin exhibited reduced antimicrobial activity against *Escherichia coli* in comparison to gentamicin. According to EUCAST 2025 [37], the clinical breakpoints for Enterobacterales are S ≤ 2 mg/L (gentamicin/tobramycin) [38]. Whether elution levels transiently exceed these thresholds depends heavily on cement matrix properties, porosity, water affinity, and assay conditions—factors which plausibly explain the observed differences between Palacos^®^ R+G and Simplex^®^ T.

These findings suggest that neither the choice of antibiotic nor the testing methodology alone can account for the observed differences in antimicrobial efficacy, thereby strongly implicating the PMMA cement formulation as the determining factor (Table 1). Notably, the observed variations in antibiotic elution and antimicrobial activity persisted across different testing methods. This interpretation is further supported by the fact that Simplex^®^ T, despite containing twice the antibiotic load, exhibited lower elution levels compared to Palacos^®^ R+G. Although Simplex^®^ is known to exhibit the lowest antibiotic release profile among bone cements [31], it remains widely utilized in spacer fabrication [49,50,51] according to its worldwide availability. The pronounced decline in antibiotic elution over time from such spacers may compromise therapeutic efficacy, potentially leading to the incomplete eradication of residual pathogens and increasing the risk of antimicrobial resistance development [50]. Tseng et al. [51] attempted to enhance vancomycin release from Simplex^®^ P cement to improve its suitability for spacer fabrication. However, our findings suggest that selecting an alternative commercially available PMMA may offer a more straightforward and effective approach to achieving sustained antibiotic release.

The observed differences in antibiotic elution between Simplex^®^ T and Palacos^®^ R+G may be partially attributed to their viscosity characteristics. Low-viscosity (LV) cements like Simplex^®^ T (Table 1) typically require higher antibiotic concentrations (2.5% for Simplex^®^ T) to achieve comparable release profiles to high-viscosity (HV) cements like Palacos^®^ R+G. This relationship could serve as an additional explanatory factor for the inferior elution and antimicrobial efficacy observed with Simplex^®^ bone cements, beyond previously discussed aspects. Simplex^®^ features a complex chemical composition, including a PMMA-styrene copolymer matrix, and undergoes gamma irradiation for sterilization—both of which may influence its hydrophilicity and hence elution behavior. According to the Simplex^®^ marketing material, the cement offers convenient application during both the LV and MV phases [52]. However, data from Fölsch et al. [53] demonstrated that Simplex^®^ P can be safely applied at viscosities comparable to Palacos^®^ R+G during the working phase. Notably, the sticky phase of Simplex^®^ P is highly variable, ranging from approximately 2.5 to 3.0 min, whereas Palacos^®^ R+G exhibits a more consistent sticky phase of 60–75 s. This variability may affect the application timing and the degree of cement intrusion. This is supported by further observations, highlighting differences in the doughing phase and intrusion behavior between the two cements [11]. Additionally, the manufacturer suggests an earlier loss of stickiness for Simplex^®^ T, occurring around 1.3–2.0 min, which may influence clinical handling and application timing. This results in a potential risk of a too-early application when Simplex ^®^ T is still in the sticky phase and not yet ready for application.

Antibiotic release from bone cement is known to be highest during the doughing phase (in which some cements can still be sticky), which represents a critical window for drug elution [30]. Premature application of the cement—such as is possible with Simplex^®^ T—may lead to excessive early antibiotic loss, thereby reducing the amount available for sustained release. This phenomenon has important clinical implications, as it may compromise the infection prophylaxis during primary fixation. Optimizing the timing of cement application is therefore essential to maximize antibiotic retention and ensure effective local antimicrobial activity.

Overall, these results are consistent with the higher and prolonged release of gentamicin from Palacos^®^ R+G observed in the elution studies, reinforcing its potential clinical advantage in infection prevention and spacer usage [54].

## 5. Conclusions

Standardizing antibiotic elution testing methods is crucial for generating reliable and comparable data which will enhance our understanding of the efficacy of antibiotic-loaded bone cements and improve clinical outcomes in managing PJIs. Palacos^®^ R+G bone cement exhibits more efficient antibiotic release kinetics compared to Simplex^®^ T, despite containing only 50% of the antibiotic load. Additionally, Palacos^®^ R+G offers an extended antibacterial activity, lasting up to 42 days, indicating its potential for better clinical outcomes in preventing and treating PJIs. Antibiotic release corresponds with the optimal application time point: this time point varies significantly for Simplex^®^ bone cements. These differences in viscosity, composition, and doughing time likely contribute to the inferior antibiotic elution and variable application behavior of Simplex^®^ T compared to Palacos^®^ R+G.

## Figures and Tables

**Table 1:** Commercially available PMMA bone cements used for testing. MMA = methyl methacrylate, DmpT = N,N-Dimethyl-p-Toluidine, HQ = hydroquinone, E141 = sodium–copper–chlorophyllin, PMMA = polymethylmethacrylate, MMA/MA-S = methyl methacrylate–styrol–copolymer, BPO = benzoyl peroxide, MA-MMA = methyl acrylate−methyl methacrylate–copolymer.

Bone Cement	Simplex^®^ T	Palacos^®^ R+G
Cement Viscosity	Low Viscosity	High viscosity
Polymer powder [g]/Liquid monomer [mL]	40:20	40:20
Monomer Components	MMA, DmpT, HQ	MMA, DmpT, HQ, E141
Polymer Combination	PMMA, MA-S, BPO	PMMA, MA-MMA, E141
Radiopacifier Contained	Barium sulfate	Zirconium dioxide
Antibiotic Contained	Tobramycin	Gentamicin
Antibiotic Amount [g] per 40 g	1.0	0.5
Antibiotic Concentration [%]	2.5	1.25

**Table 2:** Testing of minimum inhibitory concentration (MIC) for PJI-relevant germs against aminoglycoside antibiotics with a comparable spectrum of activity (+ = testing).

Antibiotics	Structure	*Staphylococcus aureus* (DSM 799)	*Staphylococcus epidermidis* (DSM 1798)	*Escherichia coli* (DSM 1576)
Gentamicin	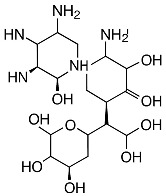	+	+	+
Tobramycin	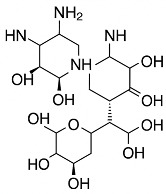	+	+	+

## Data Availability

The original contributions presented in this study are included in the article/Supplementary Material. Further inquiries can be directed to the corresponding author.

## Supplementary Materials

The following supporting information can be downloaded at https://www.mdpi.com/article/10.3390/microorganisms13092174/s1. Supplementary Data S1: Inhibition zone testing.

## Abbreviations

The following abbreviations are used in this manuscript:

ALBC	Antibiotic-loaded bone cement
ELISA	Enzyme-linked immunosorbent assay
EUCAST	European Society of Clinical Microbiology and Infectious Diseases
HPLC	High-performance liquid chromatography
HV	High-viscosity bone cement
IZT	Inhibition zone test
LV	Low-viscosity bone cement
MV	Medium-viscosity bone cement
PJI	Periprosthetic joint infection
PMMA	Polymethylmethacrylate
