# Supplementary material for: A Comparative Study of Extended Gentamicin and Tobramycin Release and Antibacterial Efficacy from Palacos and Simplex Acrylic Cements
Source: Microorganisms. 2025 Sep 17;13(9):2174. doi: 10.3390/microorganisms13092174 (PMC12473054; doi:10.3390/microorganisms13092174)
Supplement: Supplementary file 1 [file microorganisms-13-02174-s001.zip › microorganisms-3858811-supplementary.pdf]

IZT using Staphylococcus aureus

Time point	Mean (mm) Simplex T®	Mean (mm) Palacos® R+G	Adjusted p-value (Bonferroni)	Summary
1 h	16.00	17.00	>0.9999	ns
24 h	12.00	16.67	0.2505	ns
7 d	12.00	19.33	0.0808	ns
14 d	6.000	16.00	0.0336	*
21 d	6.000	16.00	0.0148	*
35 d	6.000	18.00	0.0293	*
42 d	6.000	16.00	0.0546	ns

IZT using Staphylococcus epidermidis

Time point	Mean (mm) Simplex T®	Mean (mm) Palacos® R+G	Adjusted p-value (Bonferroni)	Summary
1 h	16.00	18.00	>0.9999	ns
24 h	12.33	17.00	0.1113	ns
7 d	12.00	19.67	0.0728	ns
14 d	7.667	17.67	0.0069	**
21 d	6.667	18.00	0.0021	**
35 d	6.000	19.00	0.0080	**
42 d	6.000	18.00	0.0100	**

IZT using Echericha coli

Time point	Mean (mm) Simplex T®	Mean (mm) Palacos® R+G	Adjusted p-value (Bonferroni)	Summary
1 h	17.00	18.33	0.9351	ns
24 h	15.67	18.00	>0.9999	ns
7 d	12.33	18.67	0.2998	ns
14 d	8.000	18.33	0.0362	*
21 d	6.667	14.33	0.1404	ns
35 d	0.3333	13.00	0.0015	**
42 d	0.000	8.000	0.0362	*
